# Comprehensive advances in phytochemical components, bioactive functionality, and processing applications of mustard (*Brassica juncea* (L.) *Czern.*): a review

**DOI:** 10.3389/fnut.2025.1626333

**Published:** 2025-07-31

**Authors:** Yuao Hu, Zhengyu Yan

**Affiliations:** College of Food Science and Technology, Hunan Agricultural University, Changsha, China

**Keywords:** mustard (*Brassica juncea*), phytochemical components, functional activity, processing applications, nutrition

## Abstract

Mustard (*Brassica juncea* (L.) *Czern.*), a globally cultivated cruciferous species, is a rich source of bioactive phytochemicals, including glucosinolates (GSLs), phenolic compounds, and erucic acid (EA), which collectively contribute to its multifunctional applications in nutrition, medicine, and food processing. This review systematically elucidates the phytochemical profiles and biological activities of *B. juncea*, emphasizing structure–function relationships and processing optimization. Key phytochemical components, such as GSLs and their enzymatic degradation products, exhibit potent antioxidant, anti-inflammatory, and anticancer properties. Advanced processing techniques, including fermentation, low-sodium brining, and high-pressure treatment, are highlighted for enhancing functional compound stability and bioavailability. Despite the significant progress made, challenges still exist in understanding the genetic factors that influence phytochemical biosynthesis and in optimizing the metabolic transformations induced by processing. Future research should adopt multi-omics approaches to elucidate biosynthetic pathways, use kinetic modeling to reduce the degradation of bioactive compounds, and develop CRISPR-based strategies for improving germplasm. This comprehensive framework bridges fundamental phytochemistry with translational applications, positioning *B. juncea* as a sustainable resource for functional food innovation and precision health solutions.

## 1 Introduction

**Figure 1:**
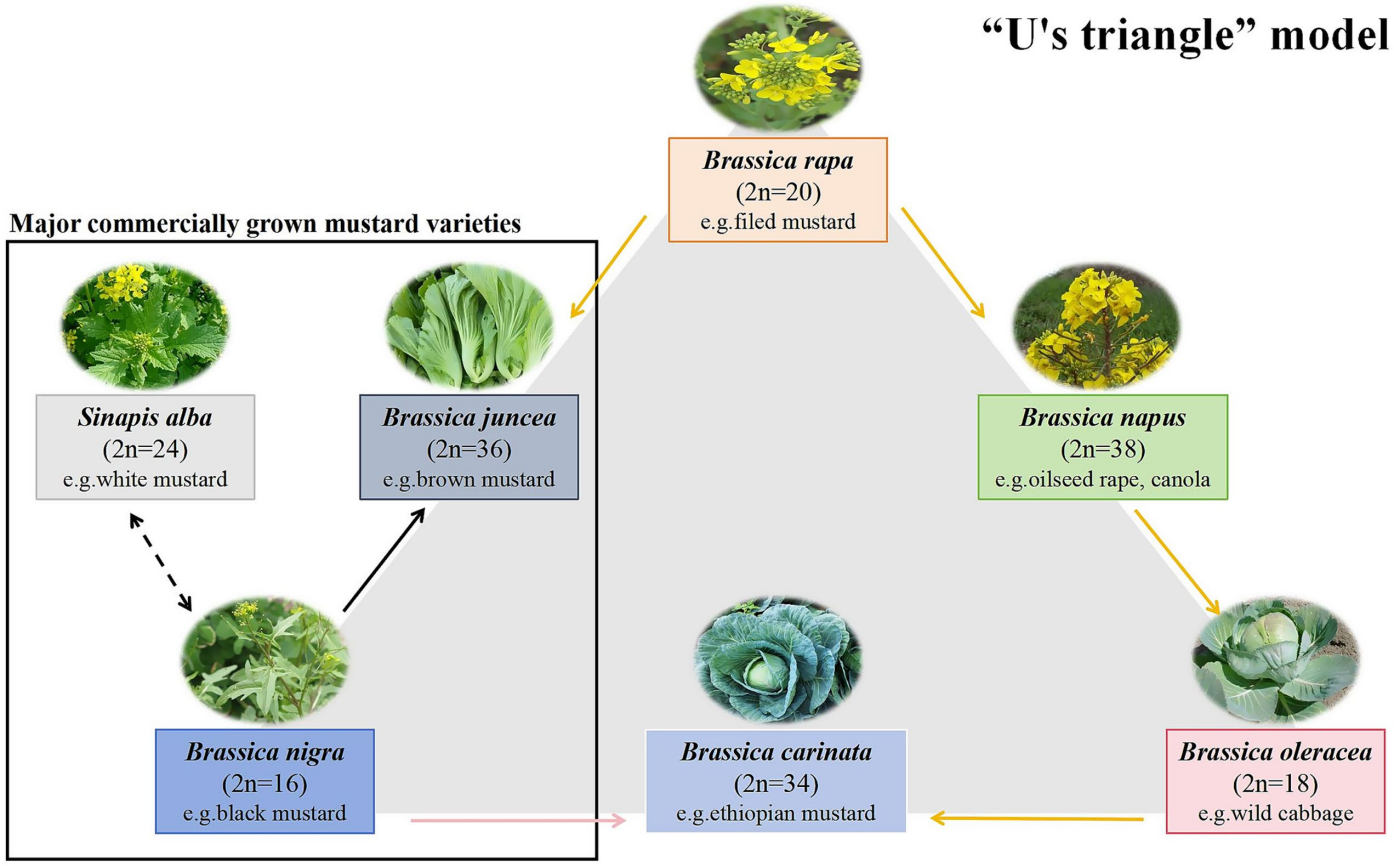
Diagram of the “U’s triangle” model. (taxa, chromosome number (n), and genetic relationships among common *Brassicaceae* species) (154, 155).

The advancing refinement of food processing technologies continues to reshape global dietary ecosystems. Currently, consumers not only pursue the organoleptic excellence, but also consider the clinically substantiated health benefits from bioactive phytochemicals in natural ingredients. This realignment has elevated functional natural foods to pivotal status within evidence-based nutritional paradigms. *B. juncea*, commonly known as Chinese mustard, Oriental mustard, or Indian mustard, is an annual herbaceous plant belonging to the genus *Brassica* within the family *Brassicaceae*. Its cultivars and related species include white mustard (*Sinapis alba*) and black mustard (*Brassica nigra*) (1, 2). The U’s triangle model shown in Figure 1 identifies some of the currently dominant mustard varieties. As a globally cultivated vegetable crop, mustard not only contains abundant proteins, vitamins, dietary fibers, and minerals but also exhibits specific metabolic characteristics for accumulating bioactive components such as glucosinolates (GSLs) and polyphenolic compounds (3, 4). Moreover, sinigrin and sinalbin are the major GSLs in mustard seeds. In *B. juncea* and *B. nigra* seeds, sinigrin is the major constituent and hydrolyzes to degrade allyl isothiocyanate (AITC), whereas the major GSLs in *S. alba* seeds is sinalbin, which hydrolyzes to produce 4-hydroxybenzyl isothiocyanate (5). These components demonstrate physiological activities, including antioxidant, anti-inflammatory, and antimicrobial properties. Influence of genetic factors, agro-ecological conditions, harvesting parameters, and post-harvest processing on the internal composition of mustard.

Notably, during fermentation, GSLs degradation products can significantly enhance these health-promoting effects (6, 7). Recent research has prioritized the functional characterization of edible components in mustard, particularly leaves and tubers. The seeds of this species, enriched with GSLs and volatile sulfides, demonstrate unique potential in flavor enhancement, natural preservative development, and agricultural biocontrol systems (8, 9). Although advancements in mustard processing technologies, including fermentation, dehydration, and bioactive compound extraction, have been explored (5, 10), several gaps still exist in the systematic integration of the following three core dimensions: the diversity of processing methods, the compositional relationships among functional constituents, and cross-domain synergistic applications.

This review systematically elaborates on the primary phytochemical components of mustard, including GSLs and their derivatives, phenolic compounds and their derivatives, as well as erucic acid (EA). It summarizes the currently prominent bioactive functions and their underlying mechanisms in mustard research, such as antioxidant, anti-cancer, anti-inflammatory, and anti-bacterial activities. Additionally, we further evaluate the latest advancements in innovative processing technologies applied to various parts of mustard, encompassing optimized cases of processes including fermentation, drying, and bioactive compound extraction. This review constructs a comprehensive theoretical framework that bridges plant taxonomy, functional food science, and agricultural product processing by integrating phytochemical characterization with mechanistic insights and technological applications. The established framework provides a multidisciplinary foundation for optimizing mustard’s culinary, therapeutic, and industrial utilization while also identifying critical knowledge gaps requiring further investigation.

## 2 Main phytochemical constituents of mustard

Mustard has been utilized as a multi-purpose crop, with cultivar-specific edible components including roots, stems, leaves, and seeds. The leaves demonstrate distinct biosynthetic capacity for chlorophyll, *β*-carotene, ascorbic acid, and essential minerals (Ca, K). This metabolic profile establishes them as superior substrates for lactic acid fermentation processes in traditional foods like sauerkraut and kimchi (11, 12). Foliar nutrient profiling reveals no significant vertical gradient in phytochemical distribution between proximal and distal leaves (13). In addition, the seeds of mustard are rich in nutrients (e.g., proteins, lipids, and carbohydrates). Genetic factors, agroecological conditions, harvest parameters, and post-harvest processing modulate Mustard’s nutritional profile. Table 1 summarizes and compares the differences in nutrient content of different mustards. In addition to nutrients, several major phytochemicals including GSLs, phenolic compounds and EA are present in mustard, which are key to the specific functional activities of mustard.

**Table 1:** Nutrients and content of major mustard varieties (g/100 g) on a dry/wet basis.

Mustard species	Component	Carbs	Protein	Dietary fiber	Calcium	Phosphorus	Iron	Vitamin	Other	Ref.
*B. juncea*	Rhizome	4.5	4.1	5.77–18.62	2.8 × 10^−1^	1.3 × 10^−1^	3.7 × 10^−3^	-	-	(157)
Indian mustard (*B. juncea*)	Seed	28	26	12	-	-	-	-	1.08 × 10^−1^ (Selenium)	(158, 159)
Pickled and dried mustard (*B. juncea, Coss*)	Whole	-	8.17 ± 0.28–3.38 ± 0.47	8.55 ± 0.97–16.68 ± 0.83	-	-	-	-	13.13 ± 0.37–21.33 ± 0.17 (Sodium)	(160)
*B. juncea* var.*gemmifera*	Whole	42.406 ± 11.51	7.595 ± 2.76	-	-	-	-	2.69 (VC)	-	(161)
Florida Broad leaf (*B. juncea* L.)	Upper leaves	-	-	-	-	3.195 × 10^−4^	-	1.314 × 10^−1^ (VC) 1.145 × 10^−4^ (VK)	-	(13)
	Lower leaves	-	-	-	-	2.61 × 10^−4^	-	1.394 × 10^−1^ (VC) 8.42 × 10^−5^ (VK)	-	
Southern Curled Giant (*B. juncea* L.)	Upper leaves	-	-	-	-	3.254 × 10^−4^	-	2.117 × 10^−1^ (VC) 1.623 × 10^−4^ (VK)	-	
	Lower leaves	-	-	-	-	5.232 × 10^−4^	-	1.196 × 10^−1^ (VC) 1.689 × 10^−4^ (VK)	-	
Mostaza negra (*B. nigra*)	Seed	38.06	20.44 ± 1.06	17.49 ± 0.66	-	-	-	-	1.26 ± 0.08 (Ash)	(162)
	Residual pasta	37.18	26.88 ± 0.01	21.13 ± 0.01	-	-	-	-	4.40 ± 0.28 (Ash)	
Mostaza amarilla (*B. alba*)	Seed	43.16	25.39 ± 0.16	15.02 ± 0.66	-	-	-	-	1.88 ± 0.08 (Ash)	
	Residual pasta	43.56	27.32 ± 0.01	17.85 ± 0.01	-	-	-	-	5.41 ± 0.35 (Ash)	
Wild mustard (*Sinapis arvensis*)	Whole	2.75 ± 0.42	1.97 ± 0.04	3.20 ± 0.09	1.233 ± 2.34 × 10^−1^	-	5.9 ± 1.18 × 10^−3^	3 × 10–4 (VA)	-	(163)

^*^Data marked as (−) were not available.

### 2.1 GSLs and their degradation products in mustard

**Figure 2:**
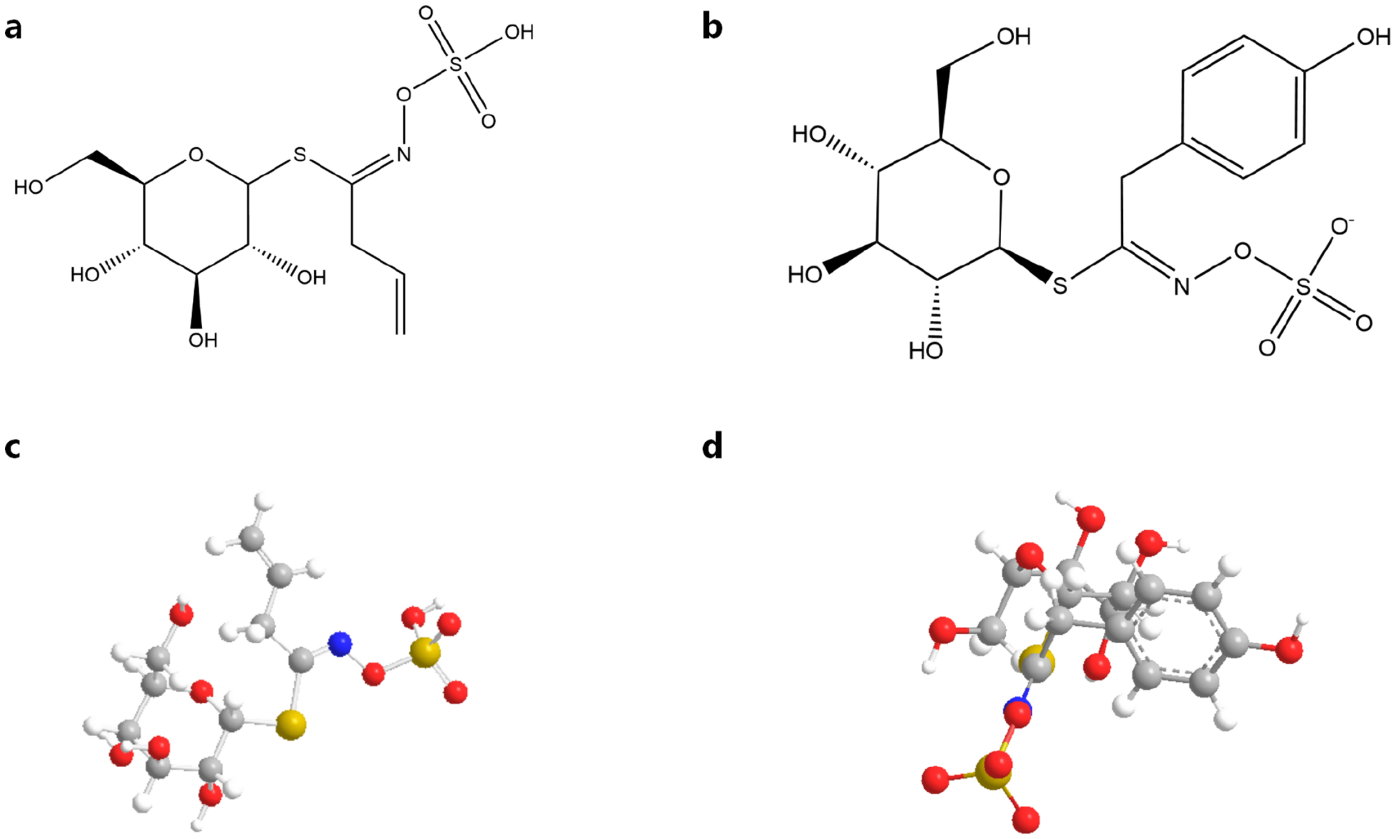
Molecular diagram of thioglucoside structure and 3-dimensional ball-and-stick model: **(a)** Sinigrin chemical molecular formula (10); **(b)** Sinalbin chemical molecular formula (10); **(c)** Sinigrin 3-dimensional ball-and-stick model; **(d)** Sinalbin 3-dimensional ball-and-stick model.

GSLs, sulfur-rich secondary metabolites in Brassicaceae species, consist of three core components: a sulfonated oxime moiety, *β*-D-glucose, and variable amino acid-derived side chains (R groups). These phytochemicals are distributed throughout mustard plants (Brassica spp.) (10, 14, 15). GSLs are classified into three structural categories based on their R group biosynthetic origins: aliphatic (methionine-derived), aromatic (phenylalanine/tyrosine-derived), and indolic (tryptophan-derived) variants (14, 15). These specialized metabolites exhibit dual functionality: as bioactive compounds with demonstrated antimicrobial and anticarcinogenic properties, and as precursors to isothiocyanates (ITCs) that determine characteristic organoleptic profiles in Brassicaceae crops (16). Figure 2 shows a molecular diagram of the thioglucoside structure and a 3-dimensional ball-and-stick model.

Myrosinase-mediated hydrolysis of GSLs generates bioactive derivatives including ITCs, nitriles, and indoles (5). This thioglucosidase is activated through cellular disruption in *Brassicaceae* plants. Its catalytic efficiency shows genotype-dependent variations, with *B. juncea* demonstrating the highest activity (2.75 U/mL), followed by *B. nigra* (1.50 U/mL) and *S. alba* (0.63 U/mL) (17). *B. nigra* myrosinase exhibits superior thermal stability compared to *S. alba* isoforms, potentially mediated by epidermal anthocyanin accumulation enhancing protein thermotolerance (17).

**Figure 3:**
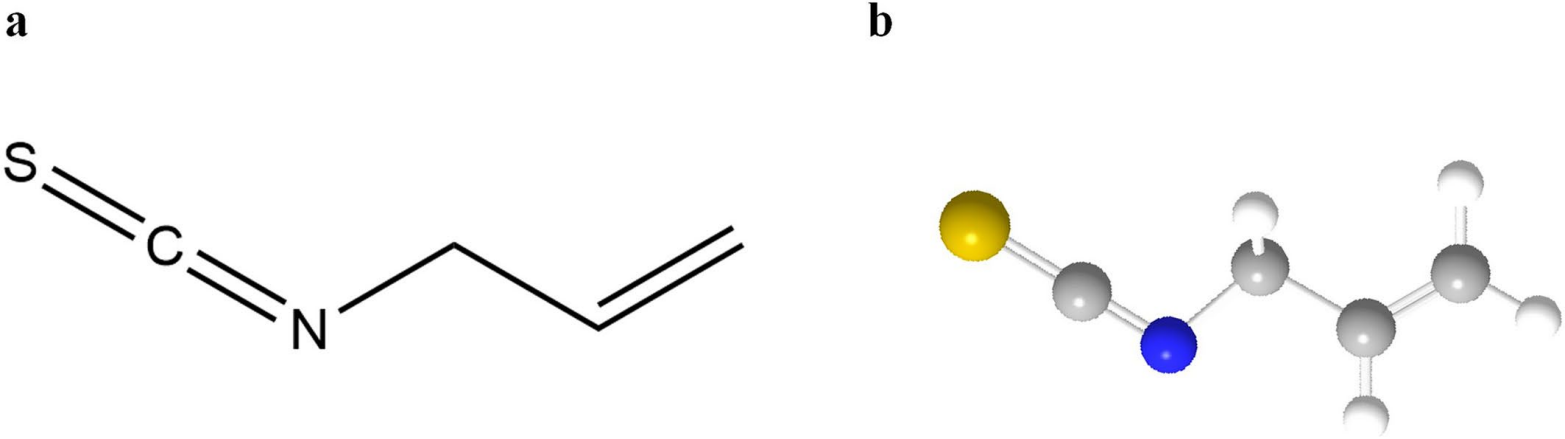
**(a)** Molecular structure of AITC; **(b)** Three-dimensional ball-and-stick model of AITC. (156).

Sinigrin is preferentially degraded in a neutral environment in the presence of Fe^2+^ to AITC (18), which is not only a core component of the pungent flavor but also exhibits broad-spectrum antimicrobial activity by disrupting the integrity of microbial membranes (19, 20). Earlier reports indicated that gavage administration of AITC was non-carcinogenic in B6C3F1 mice of both sexes, whereas an increased incidence of bladder transitional cell papillomas was observed in F344/N male rats (21). Potential mechanisms underlying the carcinogenic effects of AITC following repeated high-dose exposure in male rats have been attributed to the accumulation of its corresponding mercapturic acid conjugate. The AITC-derived mercapturic acid conjugate, N-acetyl S-(N-allylthiocarbamoyl)-L-cysteine, represents the primary urinary metabolite in both humans and rats. Notably, AITC clearance proceeds more slowly in rats compared with humans. Elevated concentrations of this N-acetylcysteine conjugate within the bladder may therefore act as a direct irritant to the bladder epithelium or dissociate into free AITC, which could similarly function as an irritant. Such irritation may induce regenerative hyperplasia and subsequent formation of benign papillomas. Given that no *in vivo* genotoxicity has been observed, it is postulated that these urinary bladder effects of AITC are mediated by a threshold mechanism (high-dose response) (5). Against this backdrop, AITC are unlikely to exert notable anti-nutritional effects in humans unless administered at high doses. Previous reports have proposed that the acceptable daily intake of AITC is 20 μg per kg of body weight, indicating no safety concerns regarding its estimated intake levels when used as a flavoring substance (5, 22). The practical application of AITC in food systems faces challenges due to its volatile nature and mucosal irritation potential. Controlled thermal processing (60–80°C, 5–15 min) modulates myrosinase activity, achieving optimal equilibrium between flavor retention and sensory acceptability (23). Figure 3 molecular diagram of the structure of AITC and a 3-dimensional ball-and-stick model.

**Figure 4:**
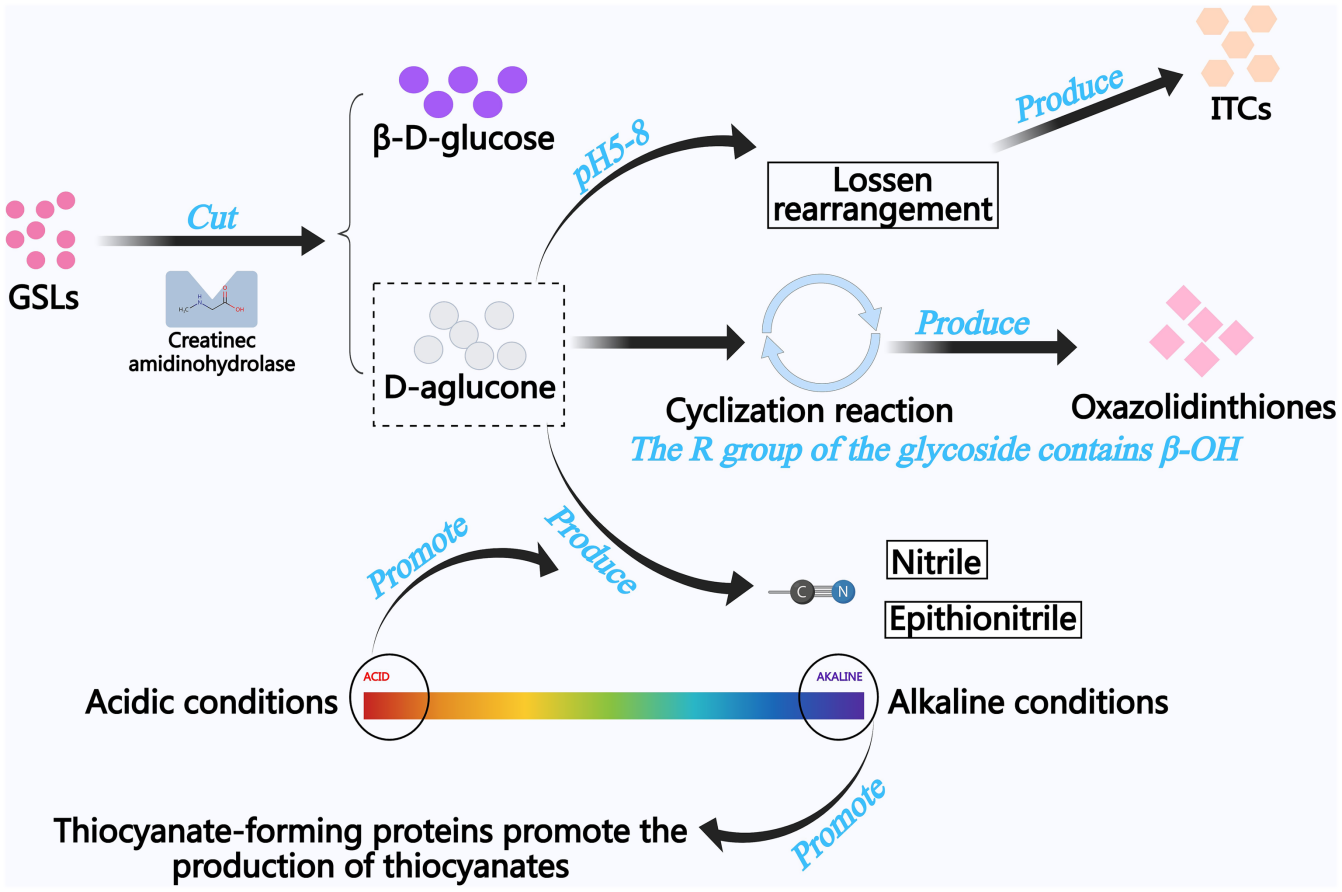
The generation process of GSLs degradation products.

The enzymatic breakdown of GSLs constitutes the central biochemical mechanism underlying flavor development in traditional fermentation processes (24). At neutral pH, ITCs are generated via Lawson rearrangement, conferring characteristic aromas to fermented products and exerting anti-inflammatory and anticancer bioactivities. (24, 25). Myrosinase-mediated thioglucosidase activity initiates GSLs cleavage, generating transient thiohydroximate-O-sulfonates. These intermediates subsequently diverge into nitriles (e.g., butyronitrile) at pH < 7 or thiocyanates at pH > 7 through distinct protonation pathways (26, 27). Figure 4 shows the generation process of GSLs degradation products.

Despite the significant health-promoting properties of ITCs, the toxicity risks associated with their degradation products (e.g., thiocyanates and nitriles) necessitate careful evaluation. Animal studies have demonstrated that chronic exposure to high doses of nitrile (>40 mg/kg/d) induces liver, kidney, and nervous system damage, whereas thiocyanate may interfere with thyroid function (28, 29). Studies employing *in vitro* models to test ITCs derived from fresh mustard juice extracts have reported partial evidence of dose-dependent genotoxic effects in bacterial and mammalian cells (30, 31). However, these early animal experimental evidences are still insufficient and human studies are limited. Instead, recent studies have focused on the anticancer properties of mustard active ingredients, particularly ITCs. The mechanism of their anticancer action is thought to be through the inhibition of cytochrome P450 enzymes, along with the induction of phase II detoxification enzymes, which prevents the activation of pro-carcinogens and promotes their binding and efflux (32). Therefore, modulation of the type and content of ITCs in mustard products by specific methods may help the active ingredients in mustard to be marketed as components of dietary supplements and concentrated herbal preparations. Current research employs precision screening of microbial consortia coupled with metabolic engineering to steer GSLs catabolism toward beneficial ITCs in fermented *Brassica* products. This metabolic flux redirection strategy maximizes ITCs yields while suppressing nitrile/thiocyanate byproducts, thereby optimizing both organoleptic qualities and nutritional integrity of fermented derivatives (27). Specific microbial taxa [*Lactiplantibacillus plantarum*, *Latilactobacillus curvatus* (33), and *Saccharomyces cerevisiae* (34)] demonstrate GSLs-modifying enzymatic capabilities. *Endophytic Bacillus* sp. NGB-B10 strains exhibit potent GSLs hydrolase activity, as characterized by Youseif et al. (35). Tuber-associated endophytes (*Bacillus amyloliquefaciens*, *Bacillus velezensis*) demonstrate halotolerant metabolism, enabling efficient GSLs bioconversion in high-salt (12–18% NaCl) fermentation systems (27).

The enzymatic degradation of sinalbin generates 4-hydroxybenzyl isothiocyanate, which contributes to the characteristic pungency of *S. alba* products and demonstrates bacteriostatic activity against *Salmonella enteritidis* and *Schizosaccharomyces pombe* (36, 37). Residual glucosinolates exceeding 2% dry mass in defatted mustard meal may disrupt thyroid function through competitive iodide uptake interference, potentially impairing livestock growth (38). Dietary glucosinolates consumed via fermented or fresh *Brassica* foods show dose-dependent chemopreventive effects, with bioactivity modulated by specific structural features of their metabolic derivatives (39, 40). GSLs derivatives exhibit concentration-dependent bioactivities, demonstrating proportional enhancement of antioxidant capacity and antimicrobial efficacy with increasing dosage (41). Subsequent research should establish structure–activity relationship models for ITCs while optimizing processing protocols to maximize functional metabolites within established safety limits (41).

### 2.2 Phenolic compounds and their derivatives in mustard

Phenolic compounds have garnered significant scientific interest for their multifaceted bioactivities, including antioxidant capacity, anti-inflammatory effects, and neuroprotective potential in functional food development (42). These phytochemicals feature ortho-diphenolic aromatic structures, systematically classified into flavonoids (quercetin, kaempferol) and non-flavonoid compounds (phenolic acids, tannins) based on hydroxylation patterns (43). *B. juncea* accumulates phenolic compounds at concentrations of 2.62–36.5 mg/g dry weight (DW), predominantly as sinapine in both free and conjugated forms (esterified or glycosylated derivatives) (44, 45).

Mustard phenolic compounds exhibit germplasm-dependent organ distribution patterns. Lateral shoots demonstrate peak accumulation (36.5 mg/g DW), followed sequentially by seeds, leaves, roots, and stems across analyzed cultivars (46, 47). Tocopherols are detectable in mustard seeds across cultivars, with concentrations varying by genetic lineage (23). Tocopherol concentrations show interspecific variation, with *B. nigra* (453.4 mg/kg) < *B. juncea* (602.1 mg/kg) < *S. alba* (886.95–952.2 mg/kg) (8). *B. juncea* var. *Gemmifera* accumulates 395.33 ± 2.89 mg QE/g flavonoids, while Chinese leaf mustard (*B. juncea Coss*) produces 2,893 μg/g of kaempferol-3-O-(hydroxyferuloyl)-*β*-D-glucoside (48, 49). Comparative analysis of 41 mustard accessions revealed root-derived shoots contain 28–35% higher mean flavonoid and phenolic content compared to leaf and tuber counterparts (50). Inter-cultivar flavonoid variation reflects both genetic divergence and methodological variations in phytochemical quantification.

Post-harvest processing parameters critically influence the extraction efficiency and bioactive integrity of phenolic compounds. Ultrasonication lowers thermal requirements while increasing phenolic extraction yields by 18–22% compared to conventional methods (44). Thermal processing (120–140°C) induces tocopherol isomerization in *S. alba* L., enhancing *α*-tocopherol content by 7–9%. Concurrently, roasting (160–180°C) retards oxidative degradation through Maillard reaction product formation (51, 52). Ethanol-water (70% v/v) solvent systems demonstrate 12–15% higher polyphenol recovery versus methanol–water mixtures, attributable to enhanced hydrogen bonding with glycosylated phenolics (53, 54). Statistical modeling optimized *B. juncea* and *S. alba* seed polyphenols to 7.61 and 5.17 mg GAE/g DW, respectively, with demonstrated DPPH radical scavenging and cellular immunostimulation effects (55).

Notably, the sinapic acid derivative canolol (4-vinyl butanol), a potent antioxidant (higher activity than *α*-tocopherol), can be generated from the decarboxylation of EA by pyrolysis (160°C, 10 min), and its extraction efficiency is modulated by the solvent polarity and pretreatment (56, 57).

**Figure 5:**
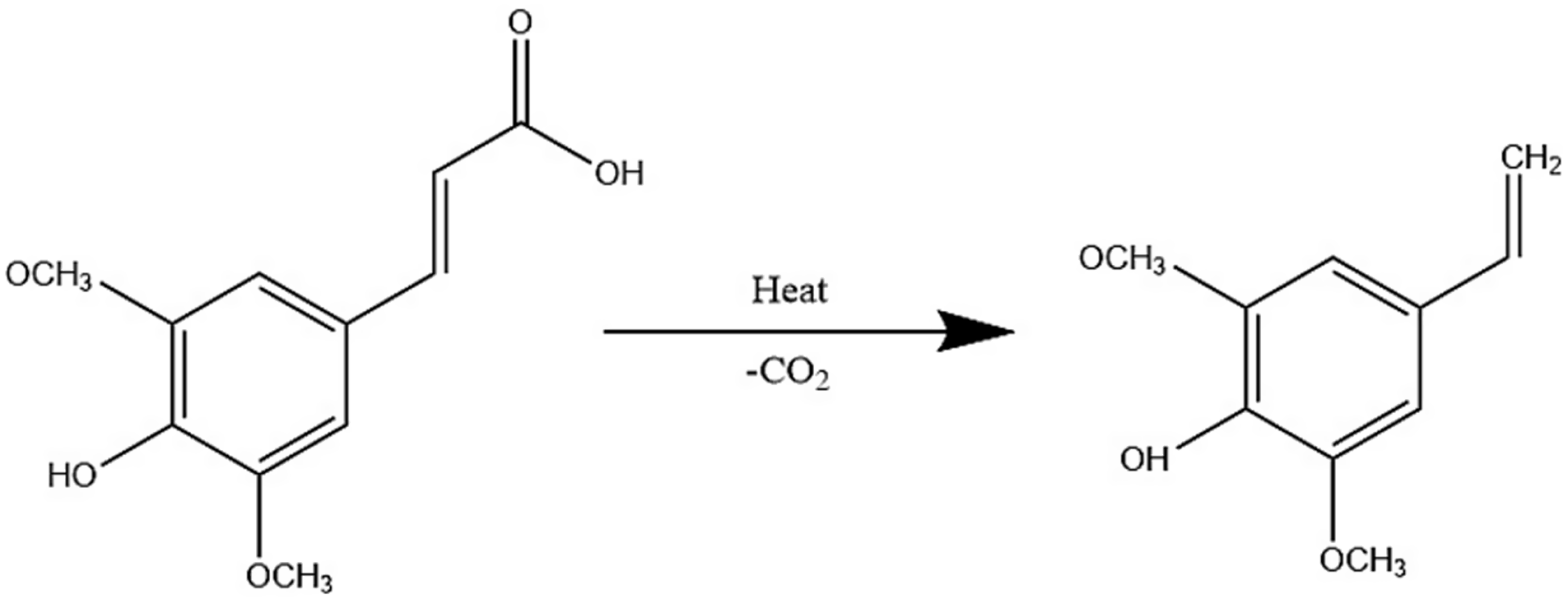
Thermal decarboxylation of mustard acid with canolol formation (57).

Figure 5 shows thermal decarboxylation of mustard acid with canolol formation. In addition, a study was conducted to optimize the extraction of phenolic compounds from mustard seeds based on electrostatic interactions between solvent and extractant using a combination of water and preheating treatments and pressure, which confirmed the extraction of sinapic acid, sinapine, and canolol in a neutral pH environment than in an acidic or alkaline environment (58, 59).

Phenolic compounds and their derivatives constitute key bioactive constituents in *Brassica* species research. Current methodologies are insufficiently characterized by their full phytochemical spectrum. Advancements in chromatographic separation and spectroscopic identification technologies, particularly high-resolution mass spectrometry coupled with metabolomic approaches, now enable comprehensive profiling of their structural diversity and metabolic interactions.

### 2.3 EA in mustard

Mustard seed serves as a significant source of edible oil and is widely utilized in the Indian subcontinent, attributed to its distinct nutty aroma, pungent flavor, and high smoke point of 250°C (60). A key distinction between mustard seed oils and other vegetable oils lies in their enrichment with long-chain monounsaturated fatty acids, including eicosenoic acid and EA (61). EA, a representative of ultra-long-chain monounsaturated fatty acids, is a 22-carbon fatty acid featuring a double bond between the C13 and C14 positions, also known as EA (C22:1). Furthermore, erucic acid constitutes the principal fatty acid in mustard seed oils from the three commercially cultivated mustard species *B. nigra*, *B. juncea*, and *S. alba*, where its levels exceed 30% of total fatty acids (5). Mustard seed oil is valued not only for its culinary properties such as its pungent flavor but also for its diverse biological activities, which are supported by experimental evidence. Epidemiological investigations in Chinese populations have revealed that breast milk from Chinese women contains the highest global concentrations of EA, and notably, the incidence rates of childhood medulloblastoma and adult glioblastoma in China remain relatively low. This correlation suggests a potential synergistic role of EA in inhibiting these tumor types (62). Mechanistic studies have gradually clarified multiple action pathways of EA. It exerts antitumor effects through bidirectional modulation of peroxisome proliferator-activated receptors (PPARs). Acting as a specific activator of PPARδ (63), it induces differentiation in C6 glioma cells and notably suppresses tumor cell clone formation and DNA synthesis capacity (64). Concurrently, EA inhibits adipocyte differentiation while promoting mesenchymal stem cell osteoblast differentiation by suppressing the transcriptional activity of PPARγ, a process that further disrupts tumor-associated metabolic reprogramming (65). In medulloblastoma models, EA further targets and inhibits the Shh signaling-driven E2F1/PPARγ axis, reducing the expression of key glycolytic enzymes (e.g., HKII, PKM2) and impairing glucose uptake efficiency, ultimately suppressing tumor cell proliferation (66). Beyond its effects via the PPARs pathway, EA also blocks tumor cell proliferation, apoptotic resistance, and the synthesis of pro-inflammatory mediators (e.g., IL-6, TNF-*α*) through inhibition of NF-κB signaling (67, 68). It further modulates the tumor microenvironment through multifaceted mechanisms, including upregulating M2-type anti-inflammatory macrophage populations and regulating lipid metabolism to lower pro-inflammatory factor levels (69). Notably, EA has also shown unique potential in the context of neurodegenerative diseases, where its role as an endogenous PPARδ ligand may attenuate neuronal damage in Huntington’s disease models via a mechanism similar to that of KD3010 (70). Animal experiments further confirmed that EA can improve the function of cholinergic system through activating key signaling pathways such as PI3K/Akt and ERK/CREB in the hippocampus, and significantly enhance the learning and memory ability of memory impairment models (71). In addition, erucamide, a derivative of EA, can exhibit antidepressant-like activity by modulating the hypothalamic–pituitary–adrenal axis (HPA axis) and decreasing stress hormone levels (72). Together, these mechanisms suggest that EA has potential applications in both tumor prevention and neuroprotection, but the risk of toxicity should not be overlooked (73). Animal studies have shown that high concentrations of EA can induce myocardial lipid deposition and cardiac lesions (74). Further studies revealed that peroxisomal oxidation of long-chain fatty acids such as EA inhibits the fatty acid oxidation process and is closely associated with hepatic steatosis and related metabolic disorders (75). Based on the discussion of the toxicity of EA, the Joint Food Standards Codex of Australia and New Zealand has classified EA in mustard oil as a toxic substance, specifying a maximum permissible limit of 20 g/kg in edible oils and a daily intake limit of 7.5 mg/kg of body weight (61). Beyond direct toxicity, processing methods may further exacerbate the associated health risks of mustard seed oil. Repeated frying of mustard seed oil (180°C for 10 min, repeated three times) generates lipid peroxidation products, which trigger the oxidative degradation of amino acids, non-volatile oxidized compounds, and polymeric, cyclic, or dimeric substances, ultimately forming toxic and carcinogenic compounds (76). Animal studies have confirmed that repeated frying with mustard seed oil (5 g/kg body weight, administered over 5 weeks) induces non-alcoholic fatty liver disease in rats, characterized by elevated serum levels of low-density lipoprotein cholesterol and triglycerides, reduced high-density lipoprotein cholesterol levels, and concurrent increases in hepatic enzyme activities, including alanine aminotransferase, aspartate aminotransferase, and alkaline phosphatase (77). Although epidemiological research suggests an association between high EA intake and an increased risk of gallbladder cancer, the specific underlying mechanisms remain unclear (78).

Balancing the health benefits and potential risks of mustard products hinges on controlling EA content as a critical strategy. Traditional breeding approaches have successfully developed “zero EA” mustard varieties (79), while researchers have also explored genetic modification as an alternative means to achieve this objective (80, 81). Nevertheless, scientific debates and public apprehensions regarding the safety of genetically modified technology have hindered the widespread adoption of such products. In daily diet, the proportion of EA can be reduced by blending high EA mustard seed oil with EA-free conventional edible oils (e.g., sesame oil, peanut oil, soybean oil, olive oil, etc.) to optimize the ratio of saturated fatty acids, monounsaturated fatty acids and polyunsaturated fatty acids, while preserving the bioactive substances of mustard seed oil (82). In addition, improved pre-press treatment not only improves oil yield, but also enhances nutritional value. It was found that microwave treatment for 6 min before pressing significantly increased the oil yield of mustard seed oil (associated with lower seed moisture content and more fragile tissues) and additionally reduced the average phytate and EA content, while enhancing the total antioxidant activity, *β*-carotene, and tocopherol content (83, 84). Nonetheless, commercially available mustard seeds and their oils and fats are among the foods with the highest erucic acid content.

## 3 Evaluation of the main biological activities of mustard

### 3.1 Antioxidant activity

*B. juncea* accumulates bioactive polyphenolic antioxidants that exert health benefits via radical scavenging, nitrosamine inhibition, and redox homeostasis regulation (85). Oh et al. (83, 84) demonstrated strong linear correlations between flavonoid content and antioxidant capacity in *B. juncea* leaves through ABTS radical scavenging, FRAP, and electron transfer assays. These findings establish flavonoids as primary contributors to the observed antioxidant effects.

Sinapic acid demonstrated dose-dependent antioxidant efficacy, exhibiting 33.2% DPPH radical inhibition at 20 μM. Scavenging capacity increased exponentially to 88.4% at equimolar concentrations (0.5:1) relative to reference antioxidants (86, 87). EA isolated from *Brassica* seeds displayed superior antioxidant capacity compared to sinapine and canolol derivatives in standardized ORAC assays (88). Methanolic fractions from fermented mustard extracts demonstrated the greatest nitrite scavenging efficacy, outperforming ethanolic and aqueous counterparts in simulated gastric fluid models (85, 89). Additionally, a study evaluated the antioxidant effect of ethanolic extract of mustard leaves on lipid oxidation of raw meat during storage by assaying the pH of microbial thiobarbituric acid free fatty acid system *in vitro*. The results showed that the pH of the samples tended to decrease during storage, and the thiobarbituric acid and free fatty acid contents increased significantly (*p* < 0.05). Notably, the total bacterial counts of the samples treated with 0.1 and 0.2% ethanolic extract of mustard leaves were considerably lower than those of the 0.02% ascorbic acid control group. This phenomenon suggests the potential antioxidant function of the ethanolic extract of mustard leaves during raw meat storage (90). *In vivo* studies further revealed its antioxidant potential. Mustard leaf extract (50–250 mg/kg/d) inhibited *γ*-radiation, chemically induced chromosome damage in mice, and elevated glutathione peroxidase and superoxide dismutase activities (91). Butanol extract alleviated oxidative stress by reducing blood glucose, glycosylated hemoglobin, and lipid peroxide levels in the streptozotocin-induced diabetic rat model (92).

Although numerous studies have evaluated the antioxidant capacity of mustard extracts using diverse methodologies, further research must thoroughly investigate the *in vivo* absorption kinetics, metabolic transformation pathways, and molecular mechanisms of action (e.g., modulation of the Nrf2/ARE pathway).

### 3.2 Anticancer activity

The antitumor activity of *B. juncea* is closely related to the bioactivity of the phenolic compounds and glucosamine GSLs it contains and their degradation products (93). By comparing the inhibitory effects of GSLs components in green mustard and red mustard (both belonging to *B. juncea*) from South Korea on four cancer cell lines (SNU-251, SNU-354, SNU-C4 and MCF-7), Kim et al. (94) found that red mustard exhibited significantly more vigorous antiproliferative activity against SNU-251 and SNU-C4 cell lines. Further studies showed that the degradation products of GSLs, including AITC, phenethyl isothiocyanate, sulforaphane, and benzyl isothiocyanate, exhibited specific inhibitory effects on lung cancer cells (95, 96).

Remarkably, the ethyl acetate extract of *B. juncea* var. *raya* showed broad-spectrum antitumor effects in several cancer cell models (breast cancer MCF-7/MDAMB-231, colon cancer HCT116, lung cancer A-549), with the most significant inhibitory effect on MCF-7 cells. Mechanistic studies showed that the extract induced tumor cell death via the reactive oxygen species-mediated mitochondrial apoptotic pathway, and its main active components were AITC (23%, derived from sinigrin degradation), 2-hexyl isothiocyanate (20%, derived from the parent gluconasturiin), and 3-butyl isothiocyanate (18%, degraded by gluconapin) (97, 98). The study by Tian et al. (99) further revealed that both fresh and fermented large-leaf mustard extracts induced cell cycle arrest and apoptosis in colon cancer HCT116 cells through the modulation of cell cycle-associated proteins (cyclin B, cyclin D1, and cyclin E) and pro-apoptotic factors (caspase-3 and its cleavage form), with the anticancer efficacy of the fermented product being significantly better than that of the fresh samples.

Mucilage/polysaccharide fractions produced during oil extraction of white mustard (*S. alba*) showed preventive effects against chemically induced colon cancer in an obese rat model (100). In addition, mustard seed extract inhibited epidermal Langerhans cell migration in mice by down-regulating the mRNA expression of inflammatory factors such as LFA-1, TNF-*α*, and IL-6. Its key regulatory protein, NPR1, inhibits explicitly the NF-κB signaling pathway, a family of transcription factors that play essential roles in immune regulation, tumor proliferation, and apoptosis (8, 100). Current research focuses on the active ingredients’ constitutive relationship and dosage effect to provide a theoretical basis for precisely regulating anticancer activity.

### 3.3 Antiviral, antibacterial, and anti-inflammatory activity

Mustard extract contains brassinosteroids and polyhydroxysteroids with documented antiviral properties (101). Using methanol extraction, a study was conducted to screen and evaluate the antiviral activity of 30 medicinal plants against the influenza A (H1N1) virus, alongside their cytotoxicity in MDCK cell cultures. Results demonstrated that the methanol extract of *B. juncea* effectively inhibited H1N1 virus replication across a concentration range of 0.3125–10 mg/mL and exhibited no cytotoxicity at 10 mg/mL (102). Lee et al. (101) further investigated the antiviral efficacy of *B. juncea* against the H1N1 virus using subcritical water extraction. Their findings revealed that this extract’s maximum non-toxic concentration (MNTC) was 0.5 mg/mL, at which the antiviral activity reached 50.35%, significantly exceeding that of ethanol or hot water extracts. Additionally, skimmed milk containing 0.28 mg/mL of *B. juncea* subcritical water extract achieved a 39.62% viral inhibition rate. Notably, the 0.5 mg/mL subcritical aqueous extract did not compromise cell viability, indicating its safety within the effective concentration range and confirming its significant inhibitory activity against the H1N1 virus.

The phenolic compounds isolated and characterized from *B. juncea* L. seed meal were composed of sinapic acid and its esters, with sinapine as the primary constituent. Following alkaline hydrolysis to release free sinapic acid, the phenolic fraction exhibited significantly enhanced antimicrobial activity, with minimum inhibitory concentrations (MIC) as low as 0.1 g/L against bacteria including *Bacillus subtilis*, *Escherichia coli*, and *Listeria monocytogenes*. This extract demonstrated selective inhibition against Gram-positive and Gram-negative bacteria but showed no inhibitory effect on *Lactobacillus plantarum* (103). Another study investigated the antimicrobial activity of the aqueous extract of *B. juncea* L., which displayed significant inhibitory activity against 35 bacterial strains, including 11 *Staphylococcus aureus*, 7 *Listeria monocytogenes*, and 1 *Salmonella venetia* isolate. Notably, the aqueous extract lost its antimicrobial activity entirely after 1 year of storage. At the same time, cytotoxicity assays revealed no hemolytic effects on sheep erythrocytes, confirming its safety as a natural antimicrobial substance. This study further indicated that the aqueous extract of *B. juncea* L. may influence the flavor profile of food models (104). Therefore, when developing natural food preservatives using mustard extracts for their broad-spectrum antimicrobial properties and metabolic regulation specificity, comprehensively evaluate the impact of extract concentration on flavor and stability constraints.

Mustard extracts exhibit significant bioactivity in the field of inflammation modulation. Sinapic acid and its derivatives in mustard blocked the expression of pro-inflammatory factors such as nitric oxide synthase, cyclooxygenase-2, tumor necrosis factor-*α*, and interleukin-1β by inhibiting NF-κB activity, which in turn mediated anti-inflammatory effects (105). Xian et al. (106) investigated the impact of *S. alba* and *B. juncea* seed ethanol extracts by acute inflammation model (12-O-tetradecanoylphorbol-acetate (TPA) and arachidonic acid (AA) induced mouse ear edema) and chronic inflammation model [multiple applications of croton oil (CO)] to compare the chemical composition and anti-inflammatory effects of ethanolic extracts of *S. alba* and *B. juncea* seeds. Results demonstrated that both extracts exhibited favorable anti-inflammatory activity in the TPA/AA-induced acute inflammation and CO-induced chronic inflammation models. Specifically, they significantly reduced ear thickness and effectively suppressed myeloperoxidase (MPO) activity in inflamed ear tissues.

Additionally, both extracts downregulated the protein and mRNA expression levels of pro-inflammatory cytokines TNF-*α* and IL-6 in the ears of TPA-treated mice. Notably, the ethanol extract from *S. alba* seeds displayed more potent anti-inflammatory effects than that from *B. juncea* seeds. Kim et al. (107) investigated the effects of the ethyl acetate and n-butanol fractions of mustard extract on the activity of lipopolysaccharide (LPS)-induced peritoneal macrophages. Results revealed that both fractions displayed notable biomodulatory properties, effectively inhibiting nitric oxide (NO) production and significantly decreasing nitrite synthesis levels. Notably, the ethyl acetate fraction derived from mustard leaves exhibited more pronounced protective effects in the LPS-stimulated cell system, with its inhibitory activity against nitrite synthesis being significantly greater than that of the corresponding n-butanol fraction. The above findings may reveal some intrinsic biological mechanisms by which mustard exerts anti-inflammatory activity.

### 3.4 Antiobesity activity

Mustard shows potential application in a healthy dietary structure for obese people. Through animal studies, Lee et al. (108) evaluated the antiobesity effects of *B. juncea* L. leaf extract (BLE). Using male Sprague–Dawley (SD) rats, they established a high-fat, hypercholesterolemic diet-induced model and administered 0, 3%, or 5% BLE treatments for 6 weeks, respectively. Results indicated that rats in the 5% BLE treatment group exhibited significantly lower weight gain compared to the control group (*p* < 0.05), along with a marked reduction in the weight of visceral adipose tissues (including mesenteric, epididymal, and total fat). Additionally, we observed a decrease in the food efficiency ratio in this group. Serological analysis showed that the BLE intervention significantly lowered triglycerides, total cholesterol, and low-density lipoprotein cholesterol levels, increased high-density lipoprotein cholesterol content, and improved atherosclerosis and cardiovascular risk indices. Histopathological examination of liver tissues confirmed that BLE mitigated lipid droplet accumulation, downregulated mRNA expression of glucose-6-phosphate dehydrogenase, acetyl-CoA carboxylase, and fatty acid synthase, while upregulating expression levels of cholesterol 7*α*-hydroxylase, low-density lipoprotein receptor, and peroxisome proliferator-activated receptor α. Another study found that the levels of serum cholesterol and triglycerides decreased, while the levels of beneficial cholesterol (high-density lipoprotein) increased after adding *B. nigra* seeds to the diet of diabetic rats (108). In addition, most of the phytosterols found in mustard (including brassicasterol, campesterol, and stigmasterol) have been shown to have plasma cholesterol-lowering activity (109). It has also been reported that *B. juncea* seed intervention reduced plasma cholesterol and phospholipid levels while increasing fecal bile acids and neutral sterols in a 1,2-dimethylhydrazine-induced colon cancer model. This effect may potentially benefit obesity prevention and treatment (110).

### 3.5 Antihyperglycemic activity

Diabetes mellitus represents a growing global health challenge characterized by insufficient insulin secretion from the pancreas or the inability of produced insulin to bind to its target receptors. This impairment in insulin signaling leads to hyperglycemia, which may give rise to secondary complications that can be life-threatening in severe cases (111). Current clinical hypoglycemic therapy primarily relies on chemically synthesized hypoglycemic agents and exogenous insulin replacement. With the advancement of natural product research, developing natural products with hypoglycemic activity has emerged as a key area of focus.

The hypoglycemic potential of mustard extracts has been experimentally supported (112). One study systematically analyzed the functional components and glucose metabolism regulation activities of green mustard and red mustard (*B. juncea* var. *Integrifolia*) leaf extracts, focusing on total phenolics, total thioglycosides content, and inhibitory ability on *α*-amylase and α-glucosidase (as a key enzyme catalyzing the decomposition of complex carbohydrates into glucose, the activity of *α*-glucosidase inhibition can directly reduce blood glucose levels) (113). Results revealed that the total phenolic content and antioxidant activity of green mustard leaf extract were significantly higher than those of red mustard, whereas sinigrin, a thioglycoside constituent, accumulated more abundantly in red mustard leaves. Further enzyme inhibition assays demonstrated that red mustard leaf extract exhibited more potent inhibitory activity against α-glucosidase, while its effects on α-amylase activity were relatively limited. These findings confirm its superior efficacy in blood glucose regulation compared to green mustard (114). Additionally, researchers have reported that seed extracts of *B. juncea* and *B. nigra* exhibit antidiabetic properties, including reducing blood glucose levels and enhancing glucose tolerance in animal models of diabetes (108, 115). The fiber content of diets, particularly soluble fiber, also influences blood glucose and insulin levels (116). Studies have further demonstrated that mustard mucilage (a soluble fiber) with varying dietary concentrations exerts antidiabetic effects in experimental rats by ameliorating elevated postprandial glucose levels and insulinemic status (117, 118).

Although existing studies have demonstrated the significant antihyperglycemic activity of mustard extract, the complete compositional analysis of its hypoglycemic effect and human experimental data are still insufficient, and its mechanistic details and clinical potential as a functional antidiabetic resource urgently need to be further explored.

### 3.6 Antidepressant activity

In addition to modulating the functional activity of organisms at the physiological level, mustard has shown potential ameliorative effects in mental health. In traditional applications, rapeseed oil applied via the skin is viewed as having a soothing and calming effect (119). Rahman et al. (120) developed an animal model to explore the potential antidepressant activity of the methanolic extract derived from *B. rapa* subspecies *chinensis* L. In the induced sleep latency test, mice treated with 200 mg/kg and 400 mg/kg doses of the extract exhibited significantly shortened sleep latency (*p* < 0.005) and reduced sleep duration (*p* < 0.005), indicating modulation of central nervous system excitability. Additionally, the extract notably decreased immobility time in mice during the tail suspension test (*p* < 0.05) and forced swimming test (*p* < 0.005). These findings suggest that the antidepressant effects may be associated with the regulation of monoamine neurotransmitter pathways, including 5-hydroxytryptophan (5-HT), norepinephrine (NE), and dopamine (DA). Evidence suggests that flavonoid constituents (e.g., kaempferol and isorhamnetin) are the primary bioactive components underlying these effects. Another study in a diabetic model demonstrated that BLE extract, at doses ranging from 100 to 400 mg/kg, reversed behavioral despair (manifested as reduced immobility time in the tail suspension test, *p* < 0.05) and ameliorated the learned helplessness phenotype in diabetic rats. These effects were mediated through a mechanism involving restoring monoamine neurotransmitter levels in the brain (with 5-HT, NE, and DA levels elevated by 23–133%, respectively, *p* < 0.05) and improvements in hyperglycemia and body weight. Notably, the antidepressant effects of BLE were specifically observed in the diabetic pathological context, suggesting that its antidepressant actions may arise from modulation of interactions between glucose metabolism and neurotransmitter systems (121). The above conclusions suggest that mustard or its functional components have the potential application of intervening in diabetes-related depression at the nutritional intervention level. However, the detailed analysis of its specific mechanism of action still needs to be further elucidated through subsequent experimental studies. Tables 2, 3 represent the particular cases of *in vitro* and *in vivo* experiments confirming the various biological activities of mustard, respectively.

**Table 2:** Overview of in vitro studies on the bio-functional activities of mustard.

Source	Extraction/intervention conditions	Key active components	Detection systems	Bioactive outcomes	Ref.
s*. alba* grains	Water:acetone (1:1) binary solvent extraction	3,4-Dihydroxybenzoic acid, ferulic acid	TPC (Folin–Ciocalteu), ABTS, DPPH	23-fold increase in TPC, 48-fold increase in ABTS clearance, 25-fold increase in DPPH clearance	(164)
*B. nigra* grains	Water:acetone (1:1) binary solvent extraction	3,4-Dihydroxybenzoic acid, rutinoside	TPC (Folin–Ciocalteu), ABTS, DPPH	19-fold increase in TPC, 31-fold increase in ABTS clearance, 27-fold increase in DPPH clearance	
Dolsan leaf mustard (*B. juncea*)	50% acetonitrile soxhlet extraction	Sinigrin, total phenols, total flavonoids	ABTS, EDA, FRAP	ABTS clearance 50.07%, EDA 16.67%, FRAP 110.50 mg FeSO_4_/g	(46)
Fermented mustard (*B. juncea*)	80% methanol extraction	Caffeic acid, chlorogenic acid, EGCG	DPPH, ABTS, FRAP	DPPH (EC_50_ = 9.9 mg/mL), ABTS (EC_50_ = 9.4 mg/mL), FRAP = 1,145.9 μM TE	(85)
Fermented mustard (*B. juncea*)	Water extraction	Caffeic acid, chlorogenic acid, EGCG	DPPH, ABTS, FRAP	DPPH (EC_50_ = 25.5 mg/mL), ABTS (EC_50_ = 10.5 mg/mL), FRAP = 532.9 μM TE	
Green mustard (*B. juncea*)	70% methanol extraction	Sinigrin	MTT assay	24 h/48 h inhibition rate: SNU-251(5%/10%), SNU-354(14%/11%), SNU-C4(11%/9%), MCF-7(4%/7%)	(94)
Red mustard (*B. juncea*)	70% methanol extraction	Sinigrin	MTT assay	24 h/48 h inhibition rate: SNU-251(19%/11%), SNU-354(14%/16%), SNU-C4(17%/15%), MCF-7(5%/7%)	
*B. juncea* var. *Raya* seeds	Ethyl acetate extraction	AITC, 2-hexyl isothiocyanate, 3-butyl isothiocyanate	MTT assay	MCF-7(IC_50_ = 32.93 ± 1.2 μg/mL), MDAMB-231(IC_50_ = 37.16 ± 1.8 μg/mL), PC-3(IC_50_ = 54.73 ± 2.1 μg/mL), A549(IC_50_ = 54.35 ± 1.9 μg/mL), HCT116(IC_50_ = 61.50 ± 1.7 μg/mL)	(97)
*B. juncea* var. *Raya* seeds	Dichloromethane extraction	AITC, 2-hexyl isothiocyanate, 3-butyl isothiocyanate	MTT assay	MCF-7(IC_50_ = 43.10 ± 1.5 μg/mL), MDAMB-231(IC_50_ = 51.14 ± 2.3 μg/mL), PC-3 (IC_50_ = 65.23 ± 2.5 μg/mL), A549(IC_50_ = 80.16 ± 3.1 μg/mL), HCT116(IC_50_ = 78.86 ± 2.9 μg/mL)	
Fresh leaf mustard (from Huarong County, Hunan)	70% ethanol extraction	Total soluble sugars, fat, protein, GSLs	MTT assay	24 h/48 h/72 h inhibitory effect on HCT116(IC_50_ = 190.2/125.9/120.9 μg/μL)	(99)
Fermented leaf mustard (from Huarong County, Hunan)	70% ethanol extraction	Total soluble sugars, fat, protein, GSLs	MTT assay	24 h/48 h/72 h inhibitory effect on HCT116(IC_50_ = 158.1/103.7/99.8 μg/μL)	
*B. juncea seeds*	110°C subcritical water extraction	GSLs (Sinigrin, etc.), phenolics, flavonoids, phytic acid, brassinosteroids	MTT assay, MDCK cell infection model	MNTC: 0.5 mg/mL activity retention in milk: 39.62% (0.28 mg/mL)	(101)
*B. juncea seeds*	Methanol extraction	—	MDCK cell infection model, cytotoxicity assessment	MNTC:0.3125–10 mg/mL	(102)
*B. juncea* L. seed meal	80% acetone/0.1% formic acid extracted by room temperature sonication	Sinapine, kaempferol-sinapoyl-trihexoside, sinapoyl-hexoside derivatives	UHPLC-DAD-ESI-MS/MS, MIC	*S. aureus*, *E. coli*(MIC: 0.3–0.8 g/L)	(103)
	80% acetone extract hydrolyzed by NaOH and extracted by acidification	Free sinapic acid	UHPLC-DAD-ESI-MS/MS, MIC	*B. subtilis*, *E. coli*, *L. monocytogenes*, *S. aureus* (MIC ≤0.1 g/L)	
*B. juncea* L. seeds(from India)	Methanol extraction	Phenolics, ITCs	Paper diffusion method, MIC	Gram-positive bacteria (MIC = 3.1–25 mg/mL, maximum circle of inhibition 20 mm) Gram-negative bacteria (MIC = 3.1–12.5 mg/mL, maximum circle of inhibition 20.5 mm)	(104)
*B. juncea* leaves	Methanol extraction, ethyl acetate extraction	Isorhamnetin 7-O-monoglucoside, other phenolic compounds	LPS-stimulated mouse macrophages, MTT assay	NO inhibition: 100% at 200 μg/mL (vs. control) Cell viability: Restored to 104.9% at 200 μg/mL (*p* < 0.001)	(107)
*B. juncea* leaves	Methanol extraction, ethyl acetate extraction	Isorhamnetin 3,7-di-O-β-D-glucopyranoside, other phenolic compounds	LPS-stimulated mouse macrophages, MTT assay	NO inhibition: 47% at 200 μg/mL (*p* < 0.001) Cell viability: No significant improvement	

**Table 3:** Overview of in vivo studies on the bio-functional activities of mustard.

Source	Extraction/intervention conditions	Key active components	Detection systems	Bioactive outcomes	Ref.
Leaf mustard (*B. campestris*)	Water extraction	Sinigrin, glucobrassicin, 4-methoxyglucobrassicin	Bone Marrow Micronucleus Test Lipid Peroxidation, GSH/GST/GPx Activity Assay	Significantly inhibited γ-radiation, cyclophosphamide and urea-induced chromosome damage (*p* < 0.05), enhanced SOD, GST and GPx activities, and reduced lipid peroxidation	(91)
*B. juncea* leaves	Butanol extraction	GSLs derivatives	Diabetic rat model	Blood glucose decreased by 9.5% (200 mg/kg), glycated protein decreased by 37%, superoxide anion decreased by 18%, lipid peroxidation decreased by 54% (serum)	(92)
*S. alba* seed mucus	5% (w/w) dietary intervention in SD rats/Zucker obese rats for 8 weeks	Neutral sugar, glycuronic acid	ACF count, PCNA immunohistochemistry	ACF inhibition effect: Total ACF decreased by 21% /63% in SD rats/Zucker obese rats, and large ACF decreased by 50% /60% in SD rats/Zucker obese rats	(100)
*S. alba* seeds	50% ethanol reflux extraction	Sinapin, sinalbin, myrosinase	Ear thickness measurement, MPO activity assay, ELISA, RT-PCR	TPA model: suppressed ear swelling by 63% (250 mg/kg) AA model: suppressed ear swelling by 45% (250 mg/kg) CO model: sustained suppression of chronic inflammation Significantly down-regulated TNF-α, IL-6, and IL-1*β* mRNA expression	(106)
*B. juncea* seeds	50% ethanol reflux extraction	Mainly sinapine	Ear thickness measurement, MPO activity assay, ELISA, RT-PCR	TPA model: Suppressed ear swelling by 45% (250 mg/kg) AA model: 35% inhibition of ear swelling (250 mg/kg) Stronger inhibition of IL-1β	
*B. juncea* leaves	80% ethanol extraction	Polyphenols, flavonoids, GSLs derivatives	High-fat diet rat model	Weight gain decreased by 4.9% (p < 0.05), food utilization decreased by 8.6% (*p* < 0.05), and abdominal fat decreased by 22.5% (*p* < 0.05) in the 5% dose group	(165)
*B. nigra* seeds	80% ethanol extraction	Polyphenols, flavonoids, GSLs derivatives	Diabetic rat model`	Total cholesterol decreased by 31.9%, triglycerides decreased by 37.5%, LDL decreased by 45.6%, HDL elevated by 26.7%	(108)
*B. juncea* seeds	80% ethanol extraction	Polyphenols, flavonoids, GSLs derivatives	Rat model of colon cancer	Reduced serum cholesterol by 35.1%, liver cholesterol by 18.7%, colon cholesterol by 25.2%	(110)
*B. juncea* seed powder	Mixed with standard feed at 10%, bonded with 1% starch slurry, dried at 30°C	—	Glucose oxidase, ELISA, metabolic cage	Trend toward lower blood glucose (not statistically different), significant weight gain (*p* < 0.05 on day 70), and significant reduction in serum creatinine (p < 0.05 on day 70)	(115)
Yellow mustard mucilage	Prepared according to Weber et al. (166): 63% soluble fibers, 17.8% protein, 9.2% ash, 10% fat	—	Glucose oxidase, ELISA, metabolic cage	Insulinemia significantly increased at 45 min (*p* < 0.05); Gastric emptying delayed; Small intestinal dry matter content increased	(117)
*B. rapa* subspecies *chinensis*	Methanol extraction	Flavonoids	Sleep Test, Hanging Tail Test, Forced Swimming Test	Sleep test: prolonged sleep latency (*p* < 0.005) and shortened sleep time to 25 ± 9.54 min at 400 mg/kg dose (*p* < 0.005) Tail-hanging test: decreased immobility time by 34.66 s at 400 mg/kg dose (*p* < 0.005) Forced swimming test: decreased immobility time by 89.65 s at 400 mg/kg dose (*p* < 0.005)	(120)
*B. juncea* L.	Soxhlet extraction in aqueous ethanol	Contains kaempferol, isorhamnetin, sinapic acid	Behavioral Despair Test, Learned Helplessness Test, Hanging Tail Test, Spontaneous Activity Test, Brain Monoamine Assay	Tail suspension test: 34.66 s reduction in immobility time at 400 mg/kg dose (*p* < 0.005) Behavioral despair test: 89.65 s reduction in immobility time at 400 mg/kg dose (*p* < 0.005) Brain monoamine levels: significant recovery in 5-HT, NE, DA concentrations (*p* < 0.05)	(121)

## 4 Processing applications of mustard

### 4.1 Processing applications of mustard leaf

*B. juncea* exhibits significant industrial applicability across all morphological structures in food processing systems. Serving as the primary fermentation substrate for traditional Chinese preserved vegetables, microbial bioconversion produces bioactive secondary metabolites that elevate product phytochemical profiles (122). Fermentation systems exemplified by leaf mustard products (e.g., laotan sauerkraut) utilize synergistic interactions between endogenous flavor precursors and microbial consortia to develop distinct organoleptic profiles and biofunctional attributes (123, 124). Traditional preservation protocols comprise sequential stages including wilting, brine immersion, desalination, and microbial succession. However, environmental and chemical challenges arising from high-sodium preservation methods—particularly desalting effluent discharge and nitrosamine accumulation—emerge as critical constraints for industrial sustainability (125).

Modern biotechnological interventions have optimized conventional fermentation methodologies by addressing inherent technical limitations. Microbial consortia engineering enables targeted nitrite catabolism and flavor compound biosynthesis through strategic inoculation of defined functional strains (*L. plantarum* ZJ316), establishing sustainable fermentation paradigms (125). High-throughput microbial screening platforms facilitate rapid identification of osmotolerant strains, driving innovation in reduced-sodium fermentation protocols. Reduced-sodium LAB fermentation systems demonstrate enhanced nutraceutical profiles through bioactive compound preservation, representing critical advancements in functional food production. Industrial-scale production of *B. juncea* leaf derivatives (e.g., dehydrated mustard) enables standardized preparation of heritage cuisine formulations, including “Pork with Preserved Vegetables.” Future technological integration requires developing AI-optimized fermentation control systems that synergize artisanal practices with computational food science advancements.

### 4.2 Processing applications of mustard tuber

Zhacai, as a typical product of traditional Chinese fermented tuber mustard, has become one of the most consumed kimchi categories in the world due to its unique flavor and textural properties. The core raw material of this product is tuber mustard, and its primary production area, Fuling, Chongqing, China, processes more than 500,000 tons per year. The cultivated brand “Fuling Zhacai” has become an industry benchmark (27). The traditional production process relies on a 14–15% NaCl concentration to achieve a shelf life of 3–6 months. However, the health risks associated with high sodium intake have led to a focus on low-salt processes (126).

Current research on low salinization focuses on microbial function enhancement and process synergy optimization, and the compound application of *L. plantarum* with chitosan, inulin, and other prebiotics can enhance the acid production efficiency and maintain the product crispness, as well as effectively controlling the nitrate accumulation to achieve a stable fermentation with salinity lower than 6% (127). The construction of a mixed-strain fermentation system further promoted the process of upgrading. For example, the synergistic effect of *L. plantarum* B1 and *Saccharomyces hansenii* Y2 (mixed-strain ratio of 1:1) reduced the nitrite content to 1.05 mg/kg and significantly shortened the fermentation cycle (128). However, reduced salinity may trigger microbial ecological imbalance, and studies have shown that low-salt environments lead to deterioration of product friability and promote proliferation of spoilage microorganisms (e.g., *Pseudomonas aeruginosa and Enterobacteriaceae*) (129). More notably, decreased salinity may activate the microbial amino acid decarboxylase system, leading to excessive accumulation of biogenic amines such as histamine and tyramine, the concentrations of which can be 2–3 times higher than those of the conventional process under low-salt conditions, with potential risks of cardiovascular disease and neurotoxicity (130, 131). To address this problem, CO_2_ modified atmosphere technology realized biogenic amine control through a dual mechanism, inhibiting the metabolic activity of amine-producing bacteria such as *Psychrobacter and Halomonas* on the one hand, and down-regulating the expression levels of amine-producing genes, such as amino acid decarboxylase, amine deiminase, and amine synthase on the other hand, so that the total biogenic amine content was reduced from 161.41 mg/kg to 24.76 mg/kg which provided a guarantee for the safety of low-salt fermentation (132, 133).

Technological advancements in processing systems drive industrial modernization through enhanced automation and precision engineering. Morphometric modeling-based automated peeling systems achieve <2% residual fiber retention. Integrated deep learning visual inspection modules enable real-time cortical defect detection with 98.7% accuracy, optimizing production throughput (134, 135). The application of high-pressure processing technology (≥400 MPa) effectively delays texture degradation and expands product shelf life (136). It is noteworthy that the high-salt wastewater associated with large-scale production is prone to ecological problems such as soil pore blockage and decreased hydraulic conductivity, and the development of its treatment technology has become a key link in the sustainable development of the industry (137). Future research needs to deepen the metabolic network analysis of low-salt fermentation on the basis of maintaining the traditional flavor characteristics, promote the systematic integration of intelligent equipment and clean production technology, and realize the transformation and upgrading of the health-oriented fermented food industry.

### 4.3 Processing applications of mustard seed

As a multifunctional food ingredient, the value of mustard seed is not only reflected in the field of oil and fat processing, but also as a unique food additive due to its rich content of GSLs, antioxidant components, and characteristic flavor substances. Studies have shown that the addition of mustard seed powder to meat products can effectively delay lipid oxidation and improve sensory quality, in which the white mustard powder treatment group significantly outperformed the black and brown varieties in sensory evaluation, suggesting its potential as a nitrite replacement (138). In the fermented sausage system, the introduction of mustard seeds significantly enhanced the storage stability of the product by promoting phenolic acid production, reducing redox potential, and enhancing antioxidant activity (139, 140). Overall, the addition of mustard seeds to a product leads to better overall consumer acceptance of the product. It has been verified that mustard seed extract significantly promotes the growth of lactic acid bacteria and is effective in leading to a reduction in the number of undesirable microorganisms (141). Cho et al. (142) found through comparative experiments that the use of mustard seed extract in the production of dry-aged pork loin hams resulted in increased storage stability and improved color attributes without negatively affecting product quality. Mustard seed extract can also be used to make antimicrobial films on Bologna sausages, and the use of mustard seed extract containing GSLs was effective in reducing the level of viable *Listeria monocytogenes* on this product (143).

The application areas of mustard seed deep processing products continue to expand. Defatted mustard seed meal can be biotransformed to prepare edible biopolymer films with mechanical and barrier properties that meet the standards for food packaging materials (144). Active packaging systems developed based on volatile essential oil components (e.g., AITC) have shown unique advantages in inhibiting foodborne pathogens (145, 146). Studies have shown that mustard seeds can be made into functional foods through microbial transformation processes in addition to lipid extraction. Das et al. (147) found that the abundance of active metabolites such as polyunsaturated fatty acids in fermented mustard seeds was increased by 3.2-fold compared with the raw material, and that their probiotic functions were closely related to the modulation of intestinal flora and the activation of immune response by the multi-omics technology (147).

In terms of food matrix improvement, wheat-mustard seed composite flour (90:10, w/w) increased the protein content of bread by 5% without affecting the sensory acceptability (148). A 2.5-fold increase in protein content was observed when replacing 20% wheat flour in cookie formulations, with the 15% addition group obtaining the best sensory score (149). In addition, the synergistic effect of thiol-rich amino acids (methionine, cysteine) and antioxidants in mustard seed protein extracts can effectively inhibit hydrogen peroxide accumulation in orange juice, highlighting its promising application in the beverage industry (150).

A recent study established a protein separation process for defatted mustard meal based on ultrasound-assisted alkaline extraction-isoelectric point precipitation coupling technology (pH 4.5) it. It experimentally confirmed that when the system pH, raw material particle size, ultrasound amplitude, and processing time were optimized to 11, 375 μm, 90%, and 10 min, respectively, the protein isolate yield reached 44.87%, which was a significant improvement in the efficiency compared with that of the conventional extraction method (151). Chadni et al. (152) developed an efficient extraction system for sinigrin by integrating supercritical carbon dioxide (SC-CO_2_) pretreatment with ultrasound-assisted extraction technology, which increased the extraction efficiency by 10.13% compared with the conventional method.

Apart from processing in the food industry, mustard seeds are increasingly being utilized in the field of medicine. It has been demonstrated that mustard seed-mediated nanopreparations showed significant morphological inhibitory effects on human breast cancer MCF-7 and hepatocellular carcinoma HepG-2 cell lines in a dose-dependent response. This study provides preliminary evidence for the potential application of mustard seed-based nanomaterials in tumor adjuvant therapy and functional food development. Nevertheless, the mechanism of action still needs to be elucidated in depth by ex vivo molecular-level studies, and the technology is not yet very mature (153).

**Figure 6:**
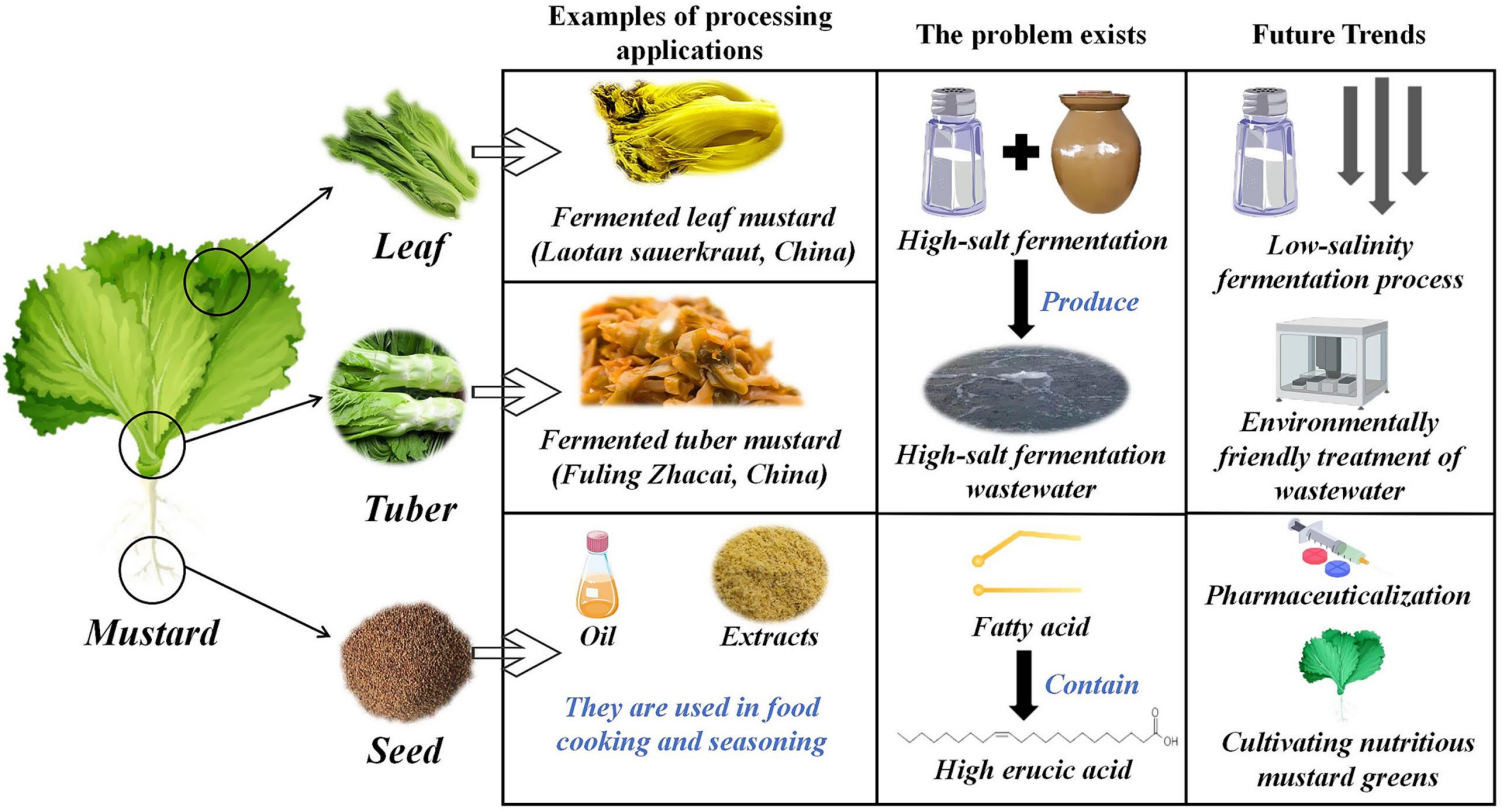
Overview of processing applications for various parts of mustard.

The industrialized processing system of mustard revolves around two core dimensions—fermentation transformation is based on multi-site specificity, and food additive development of functional components of mustard seed. Different parts of mustard, such as leaves and tubers, exhibit significant metabolite differences during fermentation. Their lactic acid bacteria-dominated microbial succession pattern directly affects the generation efficiency of product flavor substances (e.g., AITC). In contrast, the enzymatic properties of GSLs in mustard seeds provide a chemical basis for the development of natural bacteriostatic agents and flavor enhancers. With the continuous innovation of food biomanufacturing technology, microbial community regulation and optimization of fermentation kinetics for the fermentation process of mustard will become a key breakthrough point to improve product quality. In contrast, genomics-based strain-directed domestication technology is expected to modernize and upgrade the traditional process. Meanwhile, the stabilized extraction and functional characterization of the characteristic active substances in mustard seeds, as well as the expansion of their application in the fields of new preservative systems and texture improvers, will provide new solutions for the development of functional additives in the food industry. Figure 6 provides an overview of the processing applications of mustard components.

## 5 Future prospectives

Despite the gradual progress made in analyzing the phytochemical constituents of mustard, exploring its bioactivities and developing its processing applications, there is still a lot of scope for in-depth investigations. The following are some of the directions in which mustard research is evolving.

### 5.1 Elucidate molecular mechanisms

1. Integrate genomics and metabolomics to systematically clarify biosynthesis pathways and regulatory networks for GSLs, polyphenols, and EA in germplasm.
2. Analyze correlations between key gene copy number variations and metabolite accumulation to explain bioactivity differences and support targeted germplasm improvement.

### 5.2 Quantify structure–activity relationships

1. Develop quantitative models linking polyphenol structure to antioxidant/anti-inflammatory activities to reveal molecular targets and interaction mechanisms.
2. Expand in vivoand clinical trials on EA to explore its novel functional mechanisms in disease prevention/control.

### 5.3 Overcome processing bottlenecks

1. Address active ingredient loss by: (a) Revealing GSLs degradation pathways and polyphenol thermal transformation laws via metabolic flux analysis and thermodynamic modeling. (b) Optimizing fermentation processes using microbial community regulation to enhance functional ingredient retention and bioconversion efficiency.

### 5.4 Advance industrial implementation

1. Utilize multi-omics data integration to: (a) Build a mustard species-constituent-activity association database. (b) Guide targeted development of functional products (e.g., natural preservatives, functional additives). (c) Create high-activity germplasm using CRISPR. (d) Establish whole-chain QC standards covering pre-treatment, optimized processing, and storage/transport.
2. Promote the modernization and health-oriented upgrade of traditional processing towards high-value precision processing systems.

### 5.5 Foster interdisciplinary convergence

1. Leverage combined approaches (traditional knowledge and modern biotechnology) to develop mustard as a core resource for precision nutrition-oriented functional foods, laying the foundation for its large-scale application in this sector.

## 6 Conclusion

Mustard, as a functional food ingredient, contains multifaceted bioactive components, including GSLs, phenolic compounds, and erucic acid EA, which act synergistically to exert antioxidant, anticancer, anti-inflammatory, and other biological activities. However, several critical challenges remain. The variability in phytochemical composition of mustard requires careful monitoring. The potential synergistic toxicity risks associated with high-dose GSLs-derived compounds and EA need rigorous evaluation, and the hazards posed by processing by-products (e.g., nitriles) warrant thorough assessment. Additionally, the conformational relationships and cross-scale metabolic networks of these bioactive components remains limited. Although emerging processing technologies significantly influence the stability and bioavailability of active substances by modulating enzymatic reactions and metabolic pathways, the dynamic transformation of functional components during processing has not been systematically elucidated. Future studies should integrate multi-omics technologies to refine breeding strategies, couple with metabolic engineering to modulate the biosynthesis of functional constituents, and elucidate the dynamic transformation mechanisms through AI-driven intelligent manufacturing systems. Collectively, these efforts will facilitate the transformation of mustard into a precision nutrition-oriented functional food ingredient.
